# Genetic analysis of yield traits in Egyptian cotton crosses (*Gossypium barbdense* L.) under normal conditions

**DOI:** 10.1186/s12870-022-03839-8

**Published:** 2022-09-27

**Authors:** M. S. Abdel-Aty, A Youssef-Soad, W. M. B. Yehia, R. T. E. EL-Nawsany, H. M. K. Kotb, Gamal A. Ahmed, Mohamed E. Hasan, Ehab A. A. Salama, Sobhi F. Lamlom, Fouad H. Saleh, Adnan Noor Shah, Nader R. Abdelsalam

**Affiliations:** 1 Agronomy Department, Faculty of Agriculture, Kafr El-Sheikh University, Kafr el-Sheikh, 33516 Egypt; 2 Cotton Breeding Department, Cotton Research Institute, Agriculture Research Center, Giza, Egypt; 3 Plant Pathology Department, Faculty of Agriculture, Moshtohor, Benha University, Banha, Egypt; 4 Bioinformatics Department, Genetic Engineering and Biotechnology Research Institute, University of Sadat City, Sadat City, Egypt; 5 Agricultural Botany Department, Faculty of Agriculture (Saba Basha), Alexandria University, Alexandria, 21531 Egypt; 6 Plant Production Department, Faculty of Agriculture (Saba Basha), Alexandria University, Alexandria, 21531 Egypt; 7 Department of Agricultural Engineering, Khwaja Fareed University of Engineering and Information Technology, Rahim Yar Khan, 64200 Punjab Pakistan

**Keywords:** Gossypium barbadense, Heterosis, General combining ability, Specific combining ability, Line x tester

## Abstract

To generate high-yielding cultivars with favorable fiber quality traits, cotton breeders can use information about combining ability and gene activity within a population to locate elite parents and potential F1 crosses. To this end, in the current study, twelve cotton parents (eight genotypes as female parents and four testers) and their F1 crosses obtained utilizing the linex tester mating design were evaluated for their general and specialized combining abilities (GCA and SCA, respectively) of yield traits. The findings showed that for all the investigated variables, variances owing to genotypes, parents, crosses, and parent vs cross showed extremely significant (*P* ≤ 0.01) differences. Additionally, throughout the course of two growing seasons, the mean squares for genotypes (parents and crosses) showed strong significance for all the variables under study. The greatest and most desired means for all the examined qualities were in the parent G.94, Pima S6, and tester G.86. The best crossings for the qualities examined were G.86 (G.89 × G.86), G.93 × Suvin, and G.86 × Suvin. The parents' Suvin, G89x G86 and TNB were shown to have the most desired general combining ability effects for seed cotton yield/plant, lint yield/plant, boll weight, number of bolls/plants, and lint index, while Suvin, G.96 and pima S6 were preferred for favored lint percentage. For seed cotton yield, lint percentage, boll weight, and number of bolls per plant per year, the cross-G.86 x (G.89 × G.86) displayed highly significant specific combining ability impacts. The crosses G.86 × Suvin, Kar x TNB, G.93 × Suvin, and G.93 × TNB for all the studied traits for each year and their combined were found to have highly significant positive heterotic effects relative to better parent, and they could be used in future cotton breeding programs for improving the studied traits.

## Introduction

Cotton is one of the most essential multi-purposes crop due to a wide range of its benefits whether for human or animal such as feed, fiber, protein and oil. Despite the importance of cotton crop for many countries, its yield and productivity as any crop facing and affected by many environmental factors such as biotic and abiotic stresses. Therefore, the plant breeders meet a big challenge to improve cotton crop in terms of yield, yield components and fiber quality against theses harmful stresses [1]. Cotton improvement has targeted directedly towards yield and yield components characters for instance number of locules, boll size and number of bolls per plant, seeds per boll, seed size, lint index, seed index, and ginning outturn. Knowing how gene actions influence economic characteristics is essential for developing high-yielding and quality cultivars [2, 3]. Evaluating candidate lines' combining ability is not only critical for identifying superior combiner parents, but also to determine the type of gene action regulating trait inheritance [4]. Effects of combining ability are divided into general combining ability (GCA) of parents and specific combining ability (SCA) of their crosses. These effects of GCA and SCA are linked to additive and non-additive gene actions, respectively [5]. In the combining ability the entire genetic variability of each trait can be partitioned into GCA and SCA. Many authors mentioned that SCA effects are caused by genes that are non-additive (dominant or epistasis), whereas GCA effects are caused by genes that are additive in nature. They also emphasized the importance of non-additive gene activation for specific cotton characteristics [6–12]. They stressed upon the appreciable degree of variance to GCA and cleared the mean squares due to GCA and SCA were highly significant however the genetic variances due to SCA were greater than GCA for the yield traits showing the non – additive gene action [13, 14].

The main focus of the cotton improvement program was on developing hybrids, which has helped increase the productivity of cotton [15–17]. The most effective way to break yield barriers is through hybridization. It's not a good idea to choose parents based on their phenotypic performance alone, because lines with good phenotypes may produce bad recombinants in the generations that follow [18]. So, it is important that parents are chosen based on how well they can work together. Combining ability analysis is the most popular biometrical tool for finding potential parents and coming up with the best way to breed plants [19, 20].

The selection of parents or inbreeds based on their phenotypic diversity with strong combining ability is critical in developing better hybrids in a heterosis breeding program. The investigation of general and specific combining ability aids in the identification of parents or inbreeds for the generation of superior hybrids. The Line x Tester analysis is one of the easiest and most efficient methods of assessing the combining ability of many inbred/parents. Production of commercially viable hybrids is achievable based on the results of the Line x Tester analysis. Yield is a complicated polygenically inherited character that is the outcome of the multiplicative interaction of its constituent characteristics. Because it is heavily influenced by the environment, selecting solely on yield may limit future improvements. The yield component traits, on the other hand, are less complex in inheritance and are influenced by the environment to a smaller extent. Thus, selection on yield component quality can result in effective yield improvement. To differentiate between the high- and low-performing parents in a hybrid combination, data on the nature of gene activity must be evaluated. As a result, the line by tester approach aids in identifying the gene activity responsible for the manifestation of features of interest in both small and large sample sizes [21, 22]. A strategy like this also aids in the selection of prospective parents and crossing for the development of high-yielding hybrids [23]. Such a method also assists in the selection of promising parents and crosses for developing high-yielding hybrids [24–29] and provides information about GCA (additive) of parents and SCA (non-additive) of crosses, and at the same time, it helps identify the best heterotic crosses [30–33]. The most notable advantage of the line x tester technique over other crossing methods is that it requires fewer experimental materials for the mating procedure. The line x tester technique is used in cotton to study yield, its components, and fiber quality parameters [17, 34–40].

In this respect and with the above-mentioned various aspects of cotton background. The main objectives of the current research are to assess the combining ability, heterosis and performance of cotton for yield, and yield components using a line × tester mating design strategy, which helping the cotton breeder to determine best superior parents and progenies for simultaneous improvement of cotton crop for its yield, yield components and quality.

## Materials and methods

The present investigation was carried out at Sakha Agricultural Research Station during the three growing seasons 2015, 2016 and 2017. The genetic materials used in the current study were twelve genotypes, four of them as tester male parents and eight genotypes as female parents. The names, origin of these cotton genotypes is furnished in (Table 1). In 2015 season the four male testers and the eight female parents were crossed according to line x tester design to produce 32 F_1_ top crosses as out lined by [41]. The twelve genotypes were grown with their 32F_1_ hybrids for two years 2016 and 2017, respectively. The experimental design was randomized complete blocks design with three replications. Each plot was represented by one row 4 m long and 0.7 m width and 40 cm between hills and one plant were left per hill. The recommended agricultural agronomic and cultural management practices (thinning, hoeing, fertilization, irrigation etc.) by agriculture research center (ARC) were applied at the proper time as and when required.

**Table 1:** The origin and the main characters of the parents

Genotypes	Abbrev	Origin	Characteristics
G.86	G.86	Egypt	Long staple variety characterized by high yield
Karchenky	Kar	Russia	Characterized by high early maturity
G.93	G.93	Egypt	An extra-long staple variety characterized by extra fineness, strong lint, and late maturity
B.B.B	B.B.B	Greece	Long staple variety, high in boll weight and lint %
suven	Suven	India	Long staple germplasm, characterized by high lint percentage and earliness
G.89 × G.86	G.89 × G.86	Egypt	The newest long staple variety, characterized by early maturity and high number of bolls/plants
10,229 × G.86	G.94	Egypt	Characterized by fiber quality and high yielding
G.84xG70xG51 x Pima S_6_	G. 96	Egypt	Newly developed elite cotton line (extra-long)
Pima S_6_	Pima S_6_	USA	Long staple variety, characterized by high lint percentage and lint index
C.B58	C.B58	USA	A germplasm is characterized by high lint % and yield
T.N. B	T.N. B	Australia	An extra-long staple variety characterized by high lint percentage and earliness
G.70	G.70	Egypt	An extra-long staple variety characterized by high lint length 35.3 mm and Presley 11.3

At maturity stage, the data were collected and taken on the middle five plants, leaving two plants on either start or end of the row to avoid the border effects. Data were recorded on the following traits as described by [42]: Boll weight gram (B.W) was obtained from a random sample of 18 bolls collected from each plot to determine the boll weight. Seed cotton yield (g.) /plant (S.C.Y/P), It is the mean seed cotton yield harvested till final picking from the center row of each plot and expressed in grams. Lint cotton yield (g.)/plant (L.C.Y/P), It is the mean lint yield harvested and ginned till final picking from the center row of each plot and expressed in grams. Lint percentage (Ginning out turn) (L %), Seed cotton obtained from eighteen bolls for each plot chosen at random was ginned and the lint yield obtained from it was used for working out the GOT by the following formula:

$$GOT\mathrm{ \% }=\frac{weight of lint }{Weight of seed cotton }X 100$$

Seed index g. (S.I), by weighting of seed cotton (Sc) for one hundred seeds. Number of bolls/ plant (No. B./P) of random plants, Lint index (L.I), by weighting of lint from one hundred seeds.

### Statistical analysis

Statistical analysis was performed for each year. Combined analysis between the two years was done whenever homogeneity of error mean squares was detected for the studied characters according to [43]. The combining ability analysis was done using line x tester procedure as suggested by [41]. Heritability estimates were obtained as described by [44]. Path coefficient analysis was performed keeping single plant yield as the dependent variable and yield component characters with yield related traits as independent variables based on the scale of [45]. Heritability Estimates were obtained as described [38]. In R (version 3.5.2), all statistical analyses were conducted (R Core Team, 2020) where correlation diagram was performed using *corrplot* package cluster dendrogram and PCA biplot were conducted using *factoextra* package, and path analysis was performed with *lavaan* package.

The Shapiro–Wilk and Brown–Forsythe tests were used to determine whether the data had a normal distribution and whether the variances were homogeneous. Analyses of variance (ANOVA) and mean discrimination analysis were conducted on variables that met both assumptions.

## Results

### Dissecting of the relationship among parent genotypes (lines and testers)

**Fig. 1:**
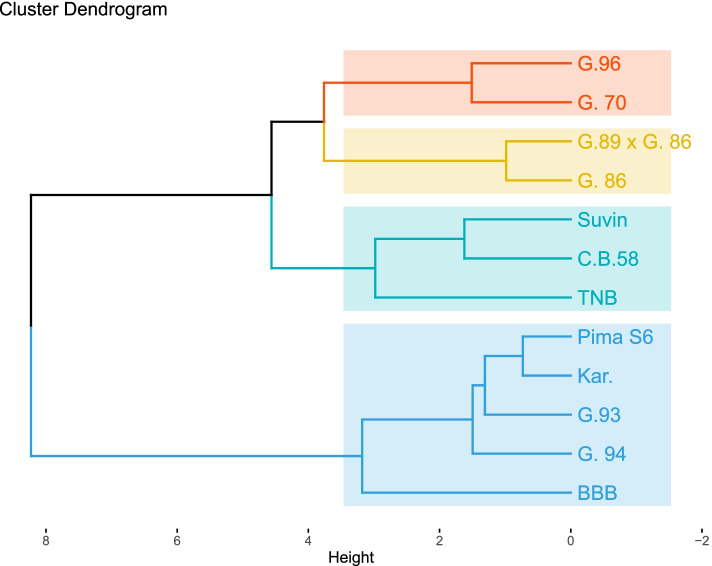
Dendrogram based on dissimilarity coefficients for yield, and yield component measured on twelve parental cotton genotypes over two years of study

The cluster analysis of six traits was conducted based on Euclidean distances using by unweighted paired group method using the arithmetic average (UPGMA). Based on the matrix data of dissimilarity coefficients, a dendrogram was performed as shown in Fig. 1. In this dendrogram, the twelve parental cotton genotypes were classified into three clusters. Cluster I included four parents and classified into two sub-clusters; (Giza 96 and Giza 70) in the first subcluster, and the other one (G.89 × G.86, and G86), while cluster II has the two closest parents Suvin and C.B58 in the first subcluster and the other one has one parent (TNB). Cluster III consisted of five parents and classified into two sub-clusters; one has four parents including Pima S6, and kar in the first sub-subcluster and Giza 93, G.94 in the second sub-subcluster while the other sub-cluster has only one parent (BBB). Genotypes grouped in the same cluster (intra cluster) are expected to be genetically more similar than genotypes grouped in different clusters (inter cluster).

### Analysis of variance

Analysis of variance (ANOVA) shown in Table 2 indicated the individual effect of growing season lines, testers factors and their interactions on six studied traits. Effect of growing season (years) was significant for all studied traits. The genotype factor affected all studied traits highly significantly. For effect of different interactions between season, lines, and testers factors, year x treatment interaction showed a highly significant effect on all studied traits. The mean squares for lines, testers and their interaction L x T were highly significant for each year and their combined over two years and this could be due to genetic diversity of parents used to generate the hybrids and environmental influences. The interaction between crosses and their partitions (L, T, and LxT) with years LxY, TxY and Lx TxY were highly significant for all the studied traits, meaning that the genotypes and their partitions affected by years.

**Table 2:** The Mean squares of twelve parents and F1 for yield and yield components traits in two years and their combined data in line X testers hybrids of cotton

S.O.V	**df**			**SCY**					**LCY**					**L%**		
	**sin**	**comb**		**Y1**		**Y2**	**comb**		**Y1**	**Y2**			**comb**	**Y1**	**Y2**	**comb**
Rep	2	-		131.25		554.54	-		40.39	48.72			-	0.56	0.42	-
Y	-	1					26,696**						3381.20**			1.14
Ea	-	4					342.75						44.55			0.49
Genotypes	43	43		12,586.74**		12,532.90**	22,756**		2086.35**	1937.85**			3714.10**	8.81**	7.67**	15.62**
Parent	11	11		4008.26**		3419.74**	6986. **		862.44**	690.86**			1491.84**	11.49**	11.52**	22.95**
Crosses	31	31		14,001.3**		11,659.5**	22,781**		2170.6**	1673.1**			3462**	5.88**	4.79**	9.51**
parent versus crosses	1	1		63,099.48**		139,853.**	195,415		12,938.3**	23,862.7**			35,971.51	70.21**	54.81**	124.54**
Lines	7	7		34,197.55**		24,148.40**	55,954**		5525.78**	3705.66**			8872.7**	13.83**	9.03**	21.88**
Testers	3	3		21,097.95**		23,551.08**	41,961*		3617.54**	3209.78**			6499.6*	14.12**	17.92**	31.75**
Lines X Testers	21	21		6255.37**		5797.73**	8984**		845.47**	776.03**			1224.6**	2.05**	1.50**	2.21
Genotypes X Y	-	43					2364**						310.1**			0.86**
Parents X Y	-	11					441.50						61.46			0.06
Crosses X Y	-	31					2879**						381.6**			1.15**
parent versus crosses X Y	-	1					7536.20**						829.43**			0.48
Lines X Y	-	7					2392**						358.8**			0.98**
Testers X Y	-	3					2688**						327.7**			0.29
Lines X Testers X Y	-	21					3069**						396.9**			1.33**
Error	86	172		129.39		460.82	295		20.90	67.42			44.2	0.29	0.38	0.34
S.O.V	**d.f**				**B.W**				**No.B.P**					**S.I**		
	**sin**		**comb**		**Y1**	**Y2**		**comb**	**Y1**		**Y2**	**comb**		**Y1**	**Y2**	**comb**
Rep	2		-		0.01	0.04		-	14.52		138.94	-		0.02	0.00	-
Y	-		1					0.02				3142.33**				9.18**
Ea	-		4					0.02				76.73				0.01
Genotypes	43		43		11.12**	0.31**		0.48**	885.56**		706.01**	1377.79**		1.60**	0.91**	2.8**
Parent	11		11		2.95**	0.25**		0.46**	198.10**		158.90**	331.78**		1.11**	1.08**	1.85**
Crosses	31		31		8.14**	0.30**		0.47**	983.25**		677.41**	1376.06**		1.54**	0.84**	1.96**
parent versus crosses	1		1		0.03**	1.40**		0.94	5419.46**		7611.03**	12,937.67		8.74**	1.34**	8.45
Lines	7		7		3.43**	0.50**		0.88**	1940.25**		894.81**	2585.89**		3.74**	1.60**	4.9**
Testers	3		3		0.61**	0.83**		0.86	1524.73**		793.90**	2180.75*		1.02**	0.90**	0.74
Lines X Testers	21		21		4.11**	0.15**		0.28**	586.90**		588.30**	857.83*		0.88**	0.60**	1.15**
Genotypes X Y	-		43					0.09**				213.77**				0.43**
Parents X Y	-		11					0.06**				25.19				0.34**
Crosses X Y	-		31					0.09**				284.59**				0.42**
parent versus crosses X Y	-		1					0.49**				92.81				1.62**
Lines X Y	-		7					0.10**				249.17**				0.43**
Testers X Y	-		3					0.18**				137.83*				1.13**
Lines X Testers X Y	-		21					0.07**				317.36**				0.32**
Error	86		172		0.87	0.02		0.01	17.34		71.61	44.47		0.05	0.05	0.05

^**a**^And ^b^: significant at 5% and 1% levels of probability, respectively. *LCY* lint cotton yield, *SCY* seed cotton yield, *SI* seed index, *BW* boll weight, *NoBP* number of bolls per plant, *L* lint percentage

### Mean performance of genotypes

The mean performance of lines, testers, and their interaction for all studied traits in each year are showed in (Table 3). The performance of genotypes appeared to be vary across years with respect to their means for all the studied traits. The results also showed that the parent G.94 had the highest and most desirable mean values for seed cotton, lint cotton yield, lint percentage, and boll weight, while the parent Pima S6 had the best desirable values for number of bolls/ Plant and seed index. On the other hand, the tester G.86 gave the highest mean values for all the studied traits except seed index, whereas the tester BBB had the highest value for the trait seed index.

**Table 3:** The mean performances of eight parental lines and four testers for yield and yield components traits in two years

	**SCY**		**LY**		**L%**		BW		NoBP		SI	
	Y1	Y2	Y1	Y2	Y1	Y2	Y1	Y2	Y1	Y2	Y1	Y2
Suvin	182.56	187.16	64.45	66.65	35.31	35.62	3.02	2.70	60.46	70.18	9.70	9.75
G.89 × G. 86	255.20	207.01	92.78	75.77	36.36	36.60	3.37	2.73	75.72	77.61	10.42	10.32
G. 94	252.81	252.35	103.85	103.55	41.06	41.03	3.52	3.40	71.72	74.13	10.13	10.29
G.96	193.01	194.27	71.69	71.65	37.14	36.88	3.22	3.06	59.95	63.59	10.97	10.19
Pima S6	220.89	228.85	77.47	79.53	35.07	34.75	2.94	2.90	75.21	78.86	10.14	11.07
C.B.58	181.16	183.09	65.53	65.87	36.16	35.97	3.15	3.14	57.55	58.26	9.79	9.32
TNB	185.03	191.98	64.23	67.07	34.73	34.94	2.91	2.77	63.51	69.47	10.11	9.66
G. 70	172.59	179.90	52.36	60.48	33.66	33.62	2.48	2.45	69.52	73.53	9.17	8.99
G. 86	278.58	288.20	104.16	108.00	37.40	37.49	3.41	3.35	81.65	85.87	11.01	10.26
Kar	239.37	245.20	84.42	86.40	35.25	35.23	3.15	3.26	75.86	75.73	10.02	9.91
G.93	175.19	198.48	59.59	67.32	33.98	33.90	2.96	2.87	59.08	69.37	9.34	9.70
BBB	222.40	234.24	80.12	84.82	36.04	36.21	3.50	3.07	63.47	76.67	10.94	10.88
LSD 0.05	18.45	34.83	7.42	13.32	0.88	1.00	0.16	0.22	6.76	13.73	0.36	0.36
LSD 0.01	24.46	46.18	9.83	17.66	1.16	1.33	0.22	0.30	8.95	18.20	0.48	0.48

*LCY* lint cotton yield, *SCY* seed cotton yield, *SI* seed index, *BW* boll weight, *NoBP* number of bolls per plant, *L* lint percentage

The mean performance of the thirty-two top crosses in each year for all the studied traits are shown in (Table 4). The results showed that the Cross G. 86 X (G.89 X G86) had the highest values for seed cotton yield/ plant, lint cotton yield/ plant and numbers of bolls/ Plant with the mean values (387.14, 151.36 and 119.50) respectively. On the other hand, the Cross-G.93 X Suvin gave the highest value for seed index with the mean value (11.55). The highest value for lint percentage (40.30) obtained from the cross-G.86 X Suvin, while the highest value for boll weight was obtained from the cross Kar. X TNB (3.59 gm).

**Table 4:** The mean performances of the thirty-two top crosses for yield and yield components traits in two years

		**SCY**		**LY**		**L%**		BW		No.B/P		SI	
Male parent	Female parents	Y1	Y2	Y1	Y2	Y1	Y2	Y1	Y2	Y1	Y2	Y1	Y2
G. 86	Suvin	352.17	347.97	144.75	137.25	41.11	39.48	3.53	3.49	99.76	99.79	11.38	10.81
	(G.89 × G. 86)	408.15	366.13	158.61	144.12	38.86	39.35	3.19	3.30	127.83	111.18	11.10	10.65
	G. 94	225.05	275.43	84.42	103.67	37.51	37.64	3.28	3.09	68.79	89.26	11.60	9.72
	G. 96	196.01	234.10	76.67	90.76	39.12	38.82	2.61	3.14	75.24	74.46	10.50	10.21
	Pima S6	277.18	300.94	110.21	118.27	39.75	39.32	3.00	2.92	92.34	103.15	10.36	10.14
	C.B.58	267.37	326.16	103.55	126.18	38.73	38.68	3.00	3.15	89.23	103.48	11.43	10.40
	TNB	368.04	314.31	138.56	120.35	37.65	38.29	3.40	3.31	108.35	95.24	10.59	10.31
	G.70	256.40	283.09	93.81	103.39	36.61	36.50	3.53	3.31	72.64	85.65	10.51	9.98
Kar	Suvin	349.55	368.13	128.53	135.19	36.77	36.71	3.32	3.79	105.18	97.17	11.41	11.54
	(G.89 × G. 86)	257.27	281.75	94.19	102.60	36.61	36.42	3.43	3.63	75.03	77.40	10.94	10.49
	G. 94	334.33	320.64	116.80	112.62	34.94	35.09	2.94	2.90	113.83	110.70	10.83	10.60
	G. 96	184.56	264.28	72.10	98.98	39.05	37.47	2.63	2.75	70.15	95.93	10.60	10.29
	Pima S6	264.77	329.69	99.25	123.37	37.47	37.43	3.35	3.60	79.12	91.67	10.51	10.57
	C.B.58	181.59	262.45	65.96	97.84	36.32	37.22	2.74	2.91	66.27	90.34	9.65	9.71
	TNB	322.26	390.19	118.15	139.49	36.66	35.75	3.56	3.62	90.57	108.00	10.64	10.40
	G.70	177.48	226.67	66.54	82.14	37.49	36.23	3.11	3.12	57.04	72.71	9.54	10.37
G.93	Suvin	351.00	409.95	138.82	153.33	39.55	37.41	3.42	3.58	102.62	114.46	12.21	10.89
	(G.89 × G. 86)	265.46	377.89	96.38	137.34	36.31	36.32	3.42	3.50	77.73	108.08	11.51	10.72
	G. 94	193.63	191.21	70.70	68.79	36.52	35.97	3.11	3.10	62.32	61.69	9.95	9.64
	G. 96	265.08	242.22	100.32	92.91	37.84	38.38	3.53	3.52	75.07	68.99	10.69	10.13
	Pima S6	321.23	257.74	120.75	97.16	37.59	37.68	3.34	3.44	96.36	75.08	10.75	9.78
	C.B.58	302.89	368.48	113.28	139.25	37.40	37.78	3.28	3.54	92.30	104.19	10.74	9.87
	TNB	312.13	355.49	115.07	129.88	36.87	36.55	3.31	3.33	94.85	106.78	11.11	10.47
	G.70	173.88	215.99	62.30	78.10	35.81	36.15	2.92	3.02	59.55	71.53	9.91	10.00
BBB	Suvin	320.06	306.14	127.16	121.65	39.75	39.76	3.37	3.39	95.12	90.28	11.63	10.12
	(G.89 × G. 86)	237.44	270.92	90.84	96.59	38.26	35.70	3.58	2.84	66.23	95.54	10.72	10.63
	G. 94	212.89	196.54	75.94	71.19	35.67	36.23	3.10	2.55	68.75	77.09	11.21	10.55
	G. 96	172.27	250.78	66.08	96.32	38.36	38.38	2.58	2.75	67.45	93.16	9.51	9.77
	Pima S6	177.62	177.23	69.12	69.60	38.90	39.26	2.70	2.78	65.80	63.75	9.47	8.84
	C.B.58	222.87	223.11	85.34	84.21	38.30	37.74	3.15	3.04	70.90	73.48	10.82	10.45
	TNB	282.66	267.20	103.65	101.71	36.67	38.09	3.15	3.21	89.74	83.33	11.71	10.85
	G.70	161.09	244.50	58.59	90.71	36.38	37.15	2.98	3.03	54.10	80.76	9.55	9.16
LSD 0.05		18.45	34.83	7.42	13.32	0.88	1.00	0.16	0.22	6.76	13.73	0.36	0.36
LSD 0.01		24.46	46.18	9.83	17.66	1.16	1.33	0.22	0.30	8.95	18.20	0.48	0.48

*LCY* lint cotton yield, *SCY* seed cotton yield, *SI* seed index, *BW* boll weight, *NoBP* number of bolls per plant, *L* lint percentage

### Combining ability

#### General combining ability

The average of lines x testers crosses performances were used to estimate general combining ability effects (GCA). Estimates of general combining ability effects of lines and testers for all the studied traits in each year are presented in (Table 4), Data showed that the best desirable general combining ability effects for seed cotton yield/ plant, lint cotton yield/ plant, boll weight, number of bolls/ plant and lint index were found in the parents Suvin, G.89 X G.86 and TNB. Meanwhile for favorable lint percentage were Suvin, G.96 and Pima S6, where they exhibited highly significant positive estimates of general combining ability effects. The tester G.86 exhibited highly significant positive (desirable) general combining ability effects for seed cotton yield, lint cotton yield, lint percentage, number of bolls/ plant and seed index. Also, the tester G.93 exhibited highly significant positive general combining ability effects for seed cotton yield, lint cotton yield and boll weight.

#### Specific combining ability

The SCA mean squares were significant for all studied traits. Thus, the significance of SCA (variances due to lines x testers) implied that both additive and non-additive types of variation was available for all the characters, yet additive genes were more important than the dominant genes because variance due to GCA was higher than that of SCA. Estimates of specific combining ability effects of 32 crosses for all the studied traits for each year are presented in (Tables 5 and 6). Data showed that eight, six, three, six, five and five crosses exhibited highly significant positive (desirable) effects for seed cotton yield, lint yield, lint percentage, boll weight, number of boll/ plant and seed index, respectively. The cross G.86X (G.89 X G.86) expressed high significant specific combining ability effects for seed cotton yield, lint percentage, boll weight and amount of bolls/ plant in each year and over two years. Also, the cross-G.93 X C.B58 had desirable values of specific combining ability effects for seed cotton yield, lint yield, boll weight and amount of bolls/ plant. It could be concluded that the best combiner of specific combining ability effects (Desirable) for most traits in each year and both years might be prime importance in breading program. The other crosses exhibited significant negative or insignificant negative or positive specific combining ability effects (Undesirable) for these traits (Table 6).

**Table 5:** General combining ability effects of parental genotypes for yield and yield component traits in two years

G.C.A	S.C.Y		L.Y		L%		B.W		No.B.P		S.I	
	Y1	Y2	Y1	Y2	Y1	Y2	Y1	Y2	Y1	Y2	Y1	Y2
Suvin	80.87**	69.07**	35.86**	28.57**	1.64**	0.87**	0.24**	0.36**	18.48**	10.60**	0.93**	0.59**
G.89 × G. 86	29.75**	35.19**	11.05**	11.88**	-0.14	-0.52*	0.23**	0.11**	4.51**	8.23**	0.35**	0.37**
G. 94	-20.85**	-43.03**	-11.99**	-19.22**	-1.49**	-1.23**	-0.07*	-0.30**	-3.77*	-5.14*	0.18**	-0.13*
G.96	-57.84**	-41.13**	-20.16**	-13.54**	0.94**	0.79**	-0.34**	-0.17**	-10.22**	-6.69**	-0.40**	-0.15*
Pima S6	-2.13	-22.58**	0.88	-6.18*	0.78**	0.96**	-0.08*	-0.02	1.21	-6.41*	-0.45**	-0.42**
C.B.58	-18.64**	6.07	-6.92**	3.59	0.04	0.39*	-0.13**	-0.05	-2.52*	3.05	-0.06	-0.15*
TNB	58.95**	42.82**	19.91**	14.58**	-0.69**	-0.30	0.18**	0.16**	13.68**	8.52**	0.29**	0.25**
G. 70	-70.11**	-46.41**	-28.64**	-19.69**	-1.08**	-0.10	-0.04	-0.09*	-21.36**	-12.16**	-0.84**	-0.37**
LSD 0.05	6.52	12.31	2.62	4.71	0.31	0.35	0.06	0.08	2.39	4.85	0.13	0.13
LSD 0.01	8.65	16.32	3.48	6.24	0.41	0.47	0.08	0.11	3.17	6.43	0.17	0.17
G. 86	31.47**	17.041**	14.87**	9.72**	1.02**	1.04**	0.02	0.01	9.58**	5.45**	0.21**	0.03
Kar	-3.35	16.50**	-3.76**	3.25	-0.74**	-0.93**	-0.04*	0.08**	-0.05	3.17	-0.21**	0.25**
G.93	10.84**	13.39**	3.25**	3.81*	-0.41**	-0.44*	0.12**	0.17**	0.40	-0.97	0.14**	-0.07
BBB	-38.96**	-46.93**	-14.36**	-16.78**	0.13	0.32*	-0.10**	-0.26**	-9.93**	-7.65**	-0.14**	-0.21**
LSD 0.05	4.61	8.71	1.85	3.33	0.22	0.25	0.04	0.06	1.69	3.43	0.09	0.09
LSD 0.01	6.12	11.54	2.46	4.41	0.29	0.33	0.05	0.07	2.24	4.55	0.12	0.12

^a^And ^b^significant at 5% and 1% levels of probability, respectively. *LCY* lint cotton yield, *SCY* seed cotton yield, *SI* seed index, *BW* boll weight, *NoBP* number of bolls per plant, *L* lint percentage

**Table 6:** Estimates of specific combining ability for yield and yield components in two years

		**SCY**		**LY**		**L%**		BW		No. B/P		SI	
**Male parent**	**Female parents**	**Y1**	**Y2**	**Y1**	**Y2**	**Y1**	**Y2**	**Y1**	**Y2**	**Y1**	**Y2**	**Y1**	**Y2**
G. 86	Suvin	-22.493**	-27.114*	-4.940	-9.318	0797*	0.098	0.101	-0.083	-10.486**	-6.083	-0.485**	-0.062
	(G.89 × G. 86)	84.602**	24.915*	33.736**	14.235**	0.337	1.357**	-0.231**	-0.024	31.547**	7.673	-0.183	0.003
	G. 94	-47.895**	12.437	-17.412**	4.884	0.329	0.367	0.154**	0.170*	-19.214**	-0.876	0.490**	-0.433**
	G. 96	-39.945**	-30.780*	-16.997**	-13.698**	-0.487	-0.481	-0.249**	0.096	-6.318**	-14.126**	-0.042	0.090
	Pima S6	-14.494*	17.503	-4.496	6.445	0.306	-0.149	-0.111	-0.268**	-0.641	14.287**	-0.124	0.282*
	C.B.58	-7.785	14.072	-3.354	4.590	0.032	-0.216	-0.066	-0.012	-0.018	5.152	0.558**	0.267*
	TNB	15.296*	-34.525**	4.831	-12.222*	-0.330	0.075	0.024	-0.067	2.899	-8.557	-0.634**	-0.226
	G.70	32.715**	23.493	8.631**	5.058	-0.984**	-1.051**	0.377**	0.186*	2.231	2.531	0.421**	0.080
Kar	Suvin	9.700	-6.415	-2.524	-4.914	-1.787**	-0.702	-0.050	0.148	4.554	-6.427	-0.044	0.458**
	(G.89 × G. 86)	-31.464**	-58.913**	-12.052**	-20.811**	-0.160	0.397	0.062	0.234**	-11.629**	-23.813**	0.081	-0.377**
	G. 94	96.205**	58.189**	33.594**	20.305**	-0.482	-0.213	0.1300*	-0.090	35.454**	22.844**	0.141	0.231
	G. 96	-16.571*	-0.065	-2.934	0.990	1.189**	0.132	-0.166**	-0.370**	-1.777	9.624	0.482**	-0.057
	Pima S6	7.923	64.792**	3.183	18.023**	-0.224	-0.063	0.288**	0.329**	-4.233	5.090	0.444**	0.489**
	C.B.58	-58.738**	-49.096**	-22.308**	-17.279**	-0.628*	0.294	-0.264**	-0.335**	-13.357**	-5.702	-0.804**	-0.643**
	TNB	4.333	41.897**	3.051	13.383**	0.437	-0.492	0.244**	0.167*	-5.264	6.497	-0.164	-0.349**
	G.70	-11.388	-32.388*	-0.009	-9.697*	1.656**	0.646	0.016	-0.083	-3.748	-8.113	-0.135	0.247
G.93	Suvin	-3.031	38.508**	0.760**	12.656**	0.674*	-0.493	-0.106	-0.153	1.546	15.011**	0.416**	0.115
	(G.89 × G. 86)	-37.462**	40.330**	-16.878**	13.363**	-0.790*	-0.187	0.107	0.009	-9.377**	10.997*	0.305	0.164
	G. 94	-58.683**	-68.141**	-19.515**	-24.095**	0.775*	0.169	-0.116	0.019	-16.505**	-22.025**	-1.086**	-0.422**
	G. 96	49.764**	-19.019	18.280**	-5.647	-0.337	0.551	0.578**	0.306**	2.688	-13.127**	0.229	0.090
	Pima S6	50.188**	-22.049	17.667**	-8.751	-0.421	0.304	0.122*	0.082	12.545**	-7.359	0.341	0.013
	C.B.58	48.371**	60.037**	17.990**	23.564**	0.122	0.363	0.124*	0.208	12.218**	12.292*	-0.058	-0.172
	TNB	-19.982**	10.300	-7.039**	3.206	0.320	-0.183	-0.162**	-0.201	-1.429	9.417	-0.044	0.028
	G.70	-29.170**	-39.965**	-11.265**	-14.297**	-0.344	0.084*	-0.333**	-0.271**	-1.686	-5.162	-0.102	0.184
BBB	Suvin	15.824*	-4.979	6.705*	1.576	6.705**	1.097**	0.054	0.088	4.386	-2.501	0.114	-0.512**
	(G.89 × G. 86)	-15.677*	-6.331	-4.806	-6.787	-4.806**	-1.567**	0.276**	-0.219**	-10.541**	5.143	-0.203	0.210
	G. 94	10.372	-2.485	3.333	-1.095	3.333**	-0.324	0.091	-0.099	0.265	0.057	0.456**	0.624**
	G. 96	6.752	49.864**	1.651	18.356**	1.651**	-0.202	-0.166**	-0.033	5.407*	17.674**	-0.669**	-0.123
	Pima S6	-43.617**	-42.246**	-16.354**	-15.717**	16.354**	0.516	-0.298**	-0.144	-7.672**	-12.017*	-0.661**	0.784**
	C.B.58	18.152**	-25.013*	7.671**	-10.876*	7.671**	0.441	0.207**	0.139	1.157	-11.742*	0.304*	0.548**
	TNB	0.353	-17.671	-0.844	-4.367	-0.844**	0.600	-0.106	0.101	3.794	-7.357	0.842**	0.548**
	G.70	7.839	48.861**	2.643	18.909**	2.643**	0.321	-0.060	0.167*	3.203	10.744	-0.183	-0.510**
LSD 0.05		13.05	24.63	5.24	9.42	0.62	0.71	0.12	0.16	4.78	9.71	0.26	0.26
LSD 0.01		17.30	32.65	6.95	12.49	0.82	0.94	0.15	0.21	6.33	12.87	0.34	0.34

^a^and ^b^significant at 5% and 1% levels of probability, respectively. *LCY* lint cotton yield, *SCY* seed cotton yield, *SI* seed index, *BW* boll weight, *NoBP* number of bolls per plant, *L* lint percentage

#### Estimation of heterosis

The mean due to crosses and parent vs. crosses were highly significant in F1 crosses, indicating the presence of heterosis in F1 generation (Table 2). The (parent vs. crosses) x years interaction was significant indicating that heterosis for the traits, boll weight, lint percentage, seed cotton yield, plant and lint yield/ plant were inconsistent across different years which reflects the importance of selection of crosses for each year to maximize the yielding ability. Heterosis expressed, as the percentage deviation (increase or decrease) of F_1_ mean performance from the corresponding better parent for all the studied traits are presented in (Table 7).

**Table 7:** Heterosis relative to the better parents for yield and yield components traits in the two years

	SCY		LCY		L%		BW			NoBP			SI	
	Y1	Y2	Y1	Y2	Y1	Y2	Y1	Y2	Y1		Y2	Y1		Y2
G. 86 × Suvin	26.42**	20.74**	38.96**	27.09**	9.92**	5.31**	3.52	3.98	22.18**		16.22*	3.36*		5.36**
G. 86 x (G.89 × G. 86)	46.51**	27.04**	52.27**	33.45**	3.92**	4.94**	-6.35**	-1.59	56.56**		29.48**	0.76		3.26
G. 86 × G. 94	-19.21**	-4.43	-18.95**	-4.01	-8.66**	-8.26**	-7.00**	-9.30**	-15.75**		3.95	5.33**		-5.51**
G. 86 × G. 96	-29.64**	-18.77**	-26.39**	-15.96*	4.62**	3.55**	-23.56**	-6.26	-7.85		-13.28	-4.69**		-0.39
G. 86 × Pima S6	-0.50	4.42	5.81	9.51	6.29**	4.86**	-11.93**	-12.82**	13.10**		20.13*	-5.90**		-8.38**
G. 86 × C.B.58	-4.03	13.17*	-0.58	16.84**	3.58**	3.17*	-12.22**	-5.96	9.29*		20.51*	3.78*		1.39
G. 86 × TNB	32.11**	9.06	33.03**	11.44	0.68	2.12	-0.39	-1.39	32.70**		10.91	-3.84*		0.49
G. 86 × G.70	-7.96*	-1.77	-9.93**	-4.27	-2.12	-2.65	3.52	-1.19	-11.03**		-0.26	-4.57**		-2.67
Kar. x Suvin	46.03**	50.13**	52.25**	56.48**	4.13**	3.08*	5.39*	16.36**	38.65**		28.30**	13.88**		16.52**
Kar. x (G.89 × G. 86)	0.81	14.91*	1.52	18.76*	0.70	-0.50	1.78	11.45**	-1.09		-0.26	5.06**		1.71
Kar. x G. 94	32.25**	27.06**	12.47**	8.77	-14.90**	-14.48**	-16.65**	-14.69**	50.05**		46.17**	6.91**		3.07
Kar. x G. 96	-22.90**	7.78	-14.59**	14.57	5.12**	1.59	-18.30**	-15.54**	-7.52		26.66**	-3.34*		1.01
Kar. x Pima S6	10.61**	34.45**	17.57**	42.80**	6.30**	6.24**	6.13*	10.33**	4.31		16.24	3.72*		-4.52**
Kar. x C.B.58	-24.14**	7.03	-21.86**	13.25	0.44	3.48*	-13.11**	-10.84**	-12.64**		19.29*	-3.66*		-2.02
Kar. x TNB	34.63**	59.13**	39.96**	61.46**	4.02**	1.47	12.90**	10.94**	19.39**		42.61**	5.27**		4.97**
Kar. x G.70	-25.86**	-7.56	-21.18**	-4.92	6.36**	2.82	-1.27	-4.29	-24.81**		-3.98	-4.79**		4.64*
G.93 × Suvin	92.27**	106.55**	115.37**	127.75**	12.02**	5.04**	13.36**	24.88**	69.73**		63.10**	25.92**		11.72**
G.93 x (G.89 × G. 86)	4.02	82.55**	3.87	81.27**	-0.15	-0.76	1.38	21.98**	2.66		39.26**	10.50**		3.94*
G.93 × G. 94	-23.41**	-24.23**	-31.92**	-33.57**	-11.06**	-12.35**	-11.83**	-8.91**	-13.09**		-16.79	-1.81		-6.29**
G.93 × G. 96	37.34**	22.04*	39.94**	29.67**	1.88	4.06**	9.62**	15.05**	25.23**		-0.55	-2.52		-0.59
G.93 × Pima S6	45.42**	12.63	55.86**	22.18*	7.18**	8.44**	12.73**	18.37**	28.11**		-4.80	6.08**		-11.63**
G.93 × C.B.58	67.19**	85.66**	72.86**	106.85**	3.41**	5.04**	4.34	12.51**	56.22**		50.20**	9.74**		1.75
G.93 × TNB	68.69**	79.11**	79.14**	92.93**	6.17**	4.60**	11.82**	16.40**	49.33**		53.71**	9.86**		7.94**
G.93 × G.70	-0.74	8.83	4.54	16.02	5.38**	6.65**	-1.35	5.35	-14.34**		-2.72	6.14**		3.06
BBB x Suvin	43.91**	30.70**	58.71**	43.42**	10.28**	9.80**	-3.90	10.18**	49.87**		17.74	6.28**		-1.22
BBB x (G.89 × G. 86)	-6.96	15.66*	-2.10	13.88	5.22**	-2.46	2.28	-7.80*	-12.53**		23.11*	-2.01		-2.32
BBB x G. 94	-15.79**	-22.11**	-26.88**	-31.25**	-13.13**	-11.71**	-12.11**	-25.07**	-4.13		0.55	2.47		-3.09
BBB x G. 96	-22.54**	7.06	-17.52**	13.56	3.28**	4.07**	-26.45**	-10.73**	6.27		21.50*	-13.28**		-10.20**
BBB x Pima S6	-20.13**	-24.34**	-13.73**	-17.94*	7.94**	8.42**	-22.93**	-9.64**	-12.52**		-19.17*	-13.44**		-20.10**
BBB x C.B.58	0.21	-4.75	6.53	-0.71	5.90**	4.22**	-10.09**	-3.39	11.70*		-4.16	-1.10		-3.98*
BBB x TNB	27.10**	14.07	29.38**	19.92*	1.75	5.19**	-10.09**	4.23	41.28**		8.69	7.04**		-0.30
BBB x G.70	-27.57**	4.38	-26.87**	6.95	0.93	2.60	-15.03**	-1.63	-22.18**		5.33	-12.71**		-15.80**
LSD 0.05	18.45	34.83	7.42	13.32	0.88	1.00	0.16	0.22	6.76		13.73	0.36		0.36
LSD 0.01	24.46	46.18	9.83	17.66	1.16	1.33	0.22	0.30	8.95		18.20	0.48		0.48

^a^And ^b^significant at 5% and 1% levels of probability, respectively. *LCY* lint cotton yield, *SCY* seed cotton yield, *SI* seed index, *BW* boll weight, *NoBP* number of bolls per plant, *L* lint percentage

Regarding seed cotton yield/ plant, heterosis relative to better parent indicated twelve crosses showed highly significant positive heterosis values in each year. Where G.93 × Suvin exhibited highly significant positive heterosis for SCY. For lint cotton yield, eleven crosses exhibited highly significant positive heterosis relative to better parent in each year. With respect to lint percentage sixteen crosses showed highly significant positive heterosis relative to better parent in each ear and their combined.

For boll weight seven crosses gave significant positive heterotic effects relative to better parent in each year. The 32 F_1_ crosses exhibited highly significant positive heterosis relative to better parent for number of bolls/ plants in each year. Only four crosses (G.86 × Suvin), Kar. X TNB, G.93 X (G.89 X G.86) and G.93 × G.70 had highly significant positive heterosis relative to better parent in each year. The other crosses remaining exhibited significant negative or non-significant positive or negative values for these traits. It could be concluded that most of the crosses which exhibited highly significant positive heterosis relative to better parent could be utilized in perspective cotton breeding programs for improving these traits.

#### Path analysis

**Fig. 2:**
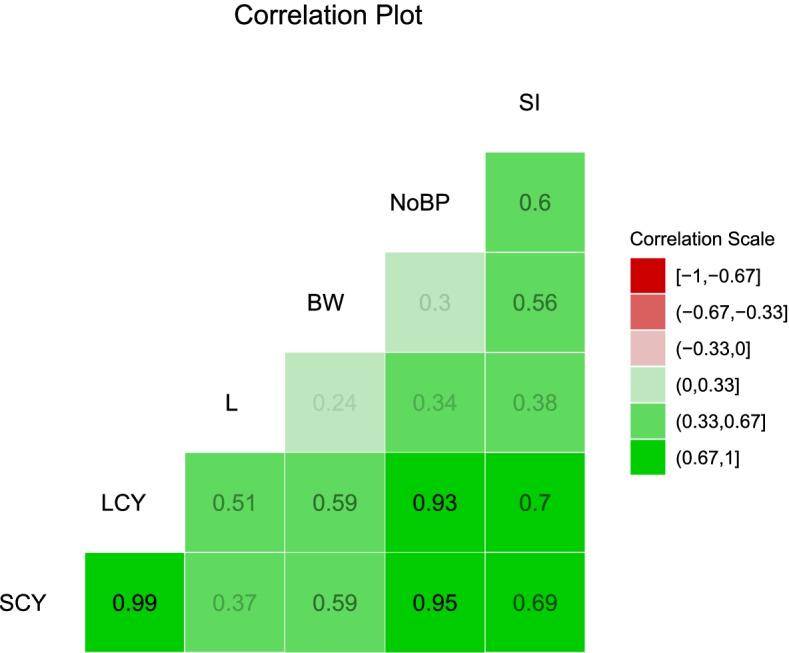
Pearson's genotypic correlation coefficients between the traits. LCY, lint cotton yield; SCY, seed cotton yield; SI, seed index; BW, boll weight; NoBP, number of bolls per plant; L, lint percentage. Crosses indicate non-significant correlations and non-crosses indicate significant correlation by t-test the 5% of probability

**Fig. 3:**
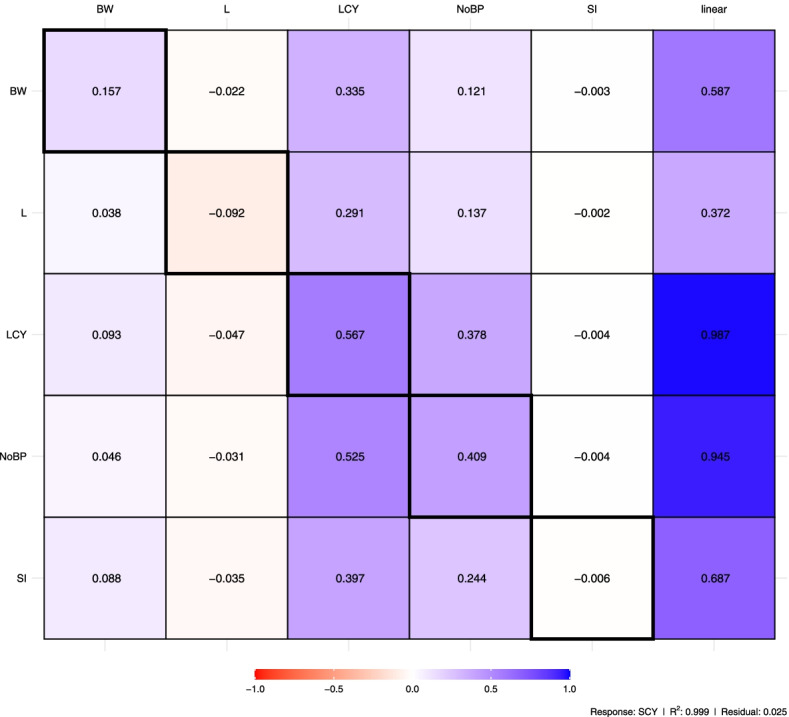
Path diagram showing the direct effect of the 6 explanatory variables on seed cotton yield examined for parents, testers and F1 crosses that evaluated over two seasons of 2016 and 2017. Bidirectional arrows show correlation between the variables, and unidirectional arrows indicate a direct effect on the direction of the arrow, blue and red arrows represent positive and negative effects. Solid arrows indicate *P* < 0.05 and dashed arrows indicate *P* > 0.05. LCY, lint cotton yield; SCY, seed cotton yield; SI, seed index; BW, boll weight; NoBP, number of bolls per plant; L, lint percentage

The result of direct and indirect correlation coefficients regressed with seed cotton yield was presented in Fig. 2 and path analysis diagram were further shown in Fig. 3. Lint yield had the highest significant positive direct effect on seed cotton yield (r = 0.99) which implied that lint yield could be used as marker for direct selection. Also, boll weight and number of bolls/plants showed significant positive direct effect on SCY (r = 0. 95 and r = 0.69). significant direct effect on SCY was recorded by BW (r = -0.59), The path coefficient analysis of indirect and direct effects of the associated traits with seed cotton yield revealed that LCY (r = 0.57) had the highest indirect contribution to seed cotton yield, followed by NOBP (r = 0.41), and BW (r = 0.16) indicating the importance of these traits to SCY. These need to be carefully considered simultaneously when selecting for yield improvement in cotton.

#### Regression analysis

**Fig. 4:**
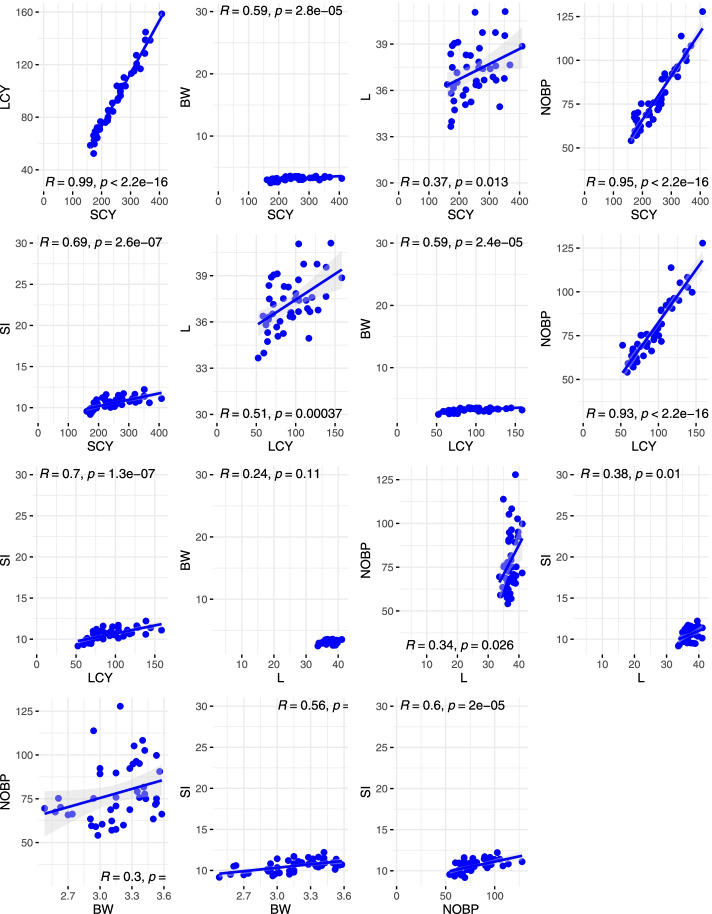
Linear regression of the yield traits, LCY, lint cotton yield; SCY, seed cotton yield; SI, seed index; BW, boll weight; No. BP, number of bolls per plant; L, lint percentage, linear mixed-effects models (second to left-most column), and log-transformed mixed-effects regression (right-most column)

Figure 4 provided the regression coefficients for all attributes and the regression coefficient of determination (R2). Figure 4 provides a graphic representation of the dependence of seed cotton yield (SCY) on key yield-related variables. The results revealed that LCY had the highest coefficient of determination (0.99), followed by No. B/P (0.95), and SI (0.69), while L percent had the lowest coefficient of determination (0.37), followed by No. B/P. (0. 59). Regression coefficient of LCY for BW, No. B/P, L percent, and SI showed that one unit's change in lint weight and boll weight caused a 59 percent, 93 percent, 50 percent, and 70 percent change (increase or decrease) in lint cotton yield (the dependent variable) (dependent variables). The range of the regression coefficient of L percent was 0.24 for BW and 0.38 for SI, respectively. In contrast, the range for BW was 0.56 (SI) to 0.3 9 (No. B/P).

## Discussion

A "line x tester" analytical approach was used to develop 32 F_1_ hybrids from a cross between eight different cotton genotypes, or "female parental lines," and four high-yielding testers in this study. Using cluster analysis, we were able to see how the different lines and testers used to build heterotic pools related in terms of the six yield attributes we were looking at. Then, we were able to cross these lines and testers. Based on this study's findings on cluster analysis, it was decided to cross twelve different female paternal genotypes and testers, resulting in the development of super heterotic hybrid cottons. As a result of this research, we can confidently say that certain cotton types can be further altered for crossbreeding utilizing cluster analysis. Previously, similar findings were found in another experiment. [6, 46–48]. Because of the wide range in combing ability among the male parents studied, the four testers were classified into two distinct clusters. Genotypic performance was used to categorize cotton genotypes into distinct groups, which may be used in cross-breeding to produce transgressive segregants in the early generations [49–53].

The results of the F1 crosses, together with the twelve parent genotypes, were examined for six yield and yield components traits. Heterosis breeding can only succeed if the genetic diversity between female parent genotypes and tester lines can be accurately measured. There were substantial genetic differences among the 44 genotypes found by using ANOVA in this investigation (*P* < 0.01) for all traits, further subsequent analysis were performed to assess combining ability [41, 54–56]. In addition, highly significant differences were exhibited across female parental genotypes, testers, and their interaction for studied traits. Practically, the combining ability of genotypes is dissected to discover genotypes with high genetic potential for developing cross combinations with desired traits and to study the activity of genes involved in trait expression [33, 57–60]. Using the Line X Tester analytic method, we can better estimate and predict essential quantitative features, which is a well-established biometrical genetics-based approach in the context of this inquiry [5, 22, 25, 54, 61–64]

Combining ability is measured via two genetic parameters, GCA and SCA, which may be respectively controlled by the additive genetic effects and non-allelic interactions of the parents [33, 65, 66]. In this investigation, positive and negative GCA effects were exhibited for different genotypes of both female parental lines and testers, indicating possessing of promising good combiner and poor combiner in term of specific traits. Female parental genotypes with strong capacity to impart desired traits to their cross offspring could be used as a significant material to improve the qualities of interest, as these genotypes have good general combining ability [9, 67, 68]. The significant GCA effects revealed in this study were consistent with previous investigations [24, 47, 69–72]. Both female parental genotypes and testers (pollinators genotypes) with positive and significant GCA effect observed in the current study are of great importance since crossing between such good combiners would result in favorable hybrid combinations in consequent segregating populations, improving selection process for specific traits. Theoretically, high GCA impact could be attributed to additive gene effects or additive x additive gene interaction effects [33, 54, 73–75]. Highly significant GCA for female parental lines and testers for LCY noticed in this study further reveal vital role of additive type of gene effects in such trait. It is noteworthy that good combiners parents for SCY were also shown to be good combiners for the majority of its yield components [76–78]. As lint yield is significantly important in such fiber crops, the female parents Suvin, G.89 X G.86 and TNB represented the best general combiner in term of LCY, revealing the most favorable genotype among female parents.

Having promising genotypes with excellent fiber quality is also urgently needed due to the increasing global demand for textile products and fierce competition from current synthetic fibers and textile industry technologies [70, 79–81]. As a result of this study, we have provided favorable crossing material for quality traits characterized with highly positive GCA effects recorded by both female parents and testers for different quality traits. This includes female parents Suvin, G.96, Pima S6 as well as G.86 and G.93 as testers for seed cotton yield, lint cotton yield, lint percentage, number of bolls/plant and seed index.

As Often, the lint yield is negatively linked with fiber quality in cotton constituting unfavorable association which has impeded cotton breeding efforts to enhance multiple fiber properties [12, 82, 83]. This may shed the light on the necessity of designing breeding programs based on such promising parents in yield traits.

Furthermore, findings of the current investigation showed that specific combining ability was highly significant for all yield traits revealing the role of non-additive gene effects as dominance or epistatic controlling these traits. One of the most promising hybrids in respect to its specific combining ability for LCY, SCY, No. B/P, and SI is G.86X (G.89 X G.86). These promising hybrids could be selected for further recombination breeding programs based on their performance and significant specific combining ability. Nevertheless, not all of F1 hybrid combinations showed positive SCA values for all the evaluated traits concurrently, stating that specific hybrid combinations having high significant SCA for several traits had both parents with a good GCA [7, 61, 84, 85].

In general, variances of GCA and SCA point out the magnitude of gene action, and this further helps in developing an appropriate breeding strategy for future breeding programs [54, 86, 87]. Variances due to GCA effects (mean squares due to lines and testers) were lower than SCA (mean squares due to lines x testers) for some traits such as LCY, SCY, SI, and NoBP indicating that the non-additive type gene action (dominant or epistatic) played an important role in governing these traits. In contrast, GCA variances were greater than SCA variances for BW, L%, and SI, which reflect the importance of additive genes against those non-additive genes controlling these traits. These findings were in consistence with those previously [8, 12, 68, 88–91].

The regression analysis was conducted to investigate the dependence between several variables. All the yield-related traits are correlated with each other in a way that increases or decreases in one trait directly affects others. Thus, estimation of association among yield,, and yield components are helpful to initiate and select the most appropriate breeding methods [92]. Estimation of phenotypic correlation among the recorded traits showed that SCY had a significant and highly positive correlation with each of NBP and LCY, indicating that selection for these two traits in yield improvement program will increase the lint yield. Similar patterns of correlation were reported in previous studies by [3, 4, 15, 22, 23, 90, 92–95]. Boll weight showed a negative correlation with NB in all three environments [96]. Likewise, significant, and positive association between BW and NSB is highly beneficial since increase in boll weight will increase the number of seeds per boll, which in turn will result in increased surface area, enhancing the maximum lint percentage. Similarly with earlier investigations, the current study found negative correlations among yield related and fiber quality traits. Correlation between SCY and LCY with fiber quality traits showed non-significant association [97–99].

Despite being essential, the correlation coefficient can lead to misunderstandings regarding the relationship between two traits and does not have to be an accurate measure of cause and effect. Thus, the strength and efficiency of the correlation coefficient between two characters may be attributed to the influence possessed by a third trait or collection of traits on the traits, which does not provide the precise relative importance of the direct and indirect effects of the elements being studied [93]. These justifications have led to using path analysis in the current study. Strikingly, the largest direct effect of SCY on the dependent variable LCY in this study implied that SCY could be used as marker to improve LCY through direct selection process. These findings were in line with recent investigations [100, 101]. Furthermore, NBP showed the highest positive indirect contribution to lint cotton yield through SCY, followed by LI through SI, BW through SI which pinpoints the importance of these traits due to their indirect vital role on improvement of LCY. Such results claim that careful and simultaneous consideration should be attained when selecting for yield improvement strategy in cotton and confirms that selection for LCY should depend on such marker traits, as well.

## Conclusion

The parent G.94 and Pima S6, as well as the tester G.86, had the best means for all the traits. The crosses G.86 (G.89 × G.86), G.93 × Suvin, and G.86 × Suvin were the elite genotype for all studied traits. The parents Suvin, G89x G86, and TNB had the most desirable GCA effects for SCY, LCY, BW, NOBP, and LI. Suvin, G.96, and pima S6 had the most desirable L%. The cross-G.86 x (G.89 × G.86) showed high significant SCA effects for SCY, L%, BW, and NOBP. whereas the crosses G.86 × Suvin, Kar x TNB, G.93 × Suvin, and G.93 × TNB had a highly significant positive heterotic effects for all the studied traits. This could be recommended this cross for use in future breeding programmes to improve both lint yield and fibre length.

## Data Availability

All the data are available in the manuscript and with Correspondence authors.
